# Unique vascular protective properties of natural products: supplements or future main-line drugs with significant anti-atherosclerotic potential?

**DOI:** 10.1186/2045-824X-4-9

**Published:** 2012-04-30

**Authors:** Mark Slevin, Nessar Ahmed, Qiuyu Wang, Garry McDowell, Lina Badimon

**Affiliations:** 1 Centro de Investigación Cardiovascular CSIC-ICCC Hospital de la Santa Creu i Sant Pau, Pavelló del Convent Sant Antoni Maria Claret, 167 08025, Barcelona, Spain; 2 School of Healthcare Science, John Dalton Building, Manchester Metropolitan University, Chester Street, Manchester, M1 5GD, UK; 3 Faculty of Health, Edge Hill University, Ormskirk, L39 4QP, UK

**Keywords:** Atherosclerotic plaque, Cardiovascular disease, Natural health products, Vascular protection

## Abstract

Natural health products (NHP) which include minerals, vitamins and herbal remedies are not generally considered by medical practitioners as conventional medicines and as such are not frequently prescribed by health centre’s as either main-line or supplemental treatments. In the field of cardiovascular medicine, studies have shown that typically, less than half of patients suffering from coronary syndromes chose to take any form of NHP supplement and these products are rarely recommended by their medical practitioner. Vascular/endothelial cell damage is a key instigator of coronary arterial plaque development which often culminates in thrombosis and myocardial infarction (MI). Current treatment for patients known to be at risk of primary or secondary (MI) includes lipid lowering statins, anti-clotting agents (e.g. tissue plasminogen activator; tPA) and drugs for stabilization of blood pressure such as beta-blockers. However, evidence has been building which suggests that components of at least several NHP (e.g. aged garlic extract (AGExt), resveratrol and green tea extracts (GTE)) may have significant vascular protective effects through reduction of oxidative stress, lowering of blood pressure, reduction in platelet aggregation, vasodilation and inhibition of abnormal angiogenesis. Therefore, in this review we will discuss in detail the potential of these substances (chosen on the basis of their potency and complimentarity) as anti-atherosclerotic agents and the justification for their consideration as main-line additional supplements or prescriptions.

## Introduction

According to a World Health Organization Fact Sheet (EURO/03/06) cardiovascular disease (CVD) is the number one killer in Europe and world-wide, with heart disease and stroke being the major cause of death in all 53 Member States. It has in fact been described as a true pandemic, with no respect for borders. Figures show that 34,421 (23% of all non-communicable diseases) of Europeans died from CVD in 2005. The report also highlighted the fact that there is approximately a 10-fold difference in premature CVD mortality between Western Europe and countries in Central and Eastern Europe (i.e. there is a higher occurrence of CVD amongst the poor and vulnerable). The problem for the European Union is that there is a direct correlation between the premature death rate and the viability of countries’ economies. Although improvements in understanding have helped to reduce the number of Western European dying from CVD and related diseases further advances will require a clearer understanding of the pathobiological mechanisms responsible for the development of atherosclerosis and myocardial infarction. Approximately 75% of acute coronary events are associated with disruption of atherosclerotic plaques, development of which takes place over several decades of life, (early vessel damage beginning during child-hood) and whose susceptibility to instability and thrombosis is largely dependent on a number of known risk-factors (dyslipidemia, arterial hypertension, hyperglycaemia and diabetes) [1].

## Key features of coronary atherosclerotic plaque development

In the early stages of vessel damage prior to plaque formation, chronic minimal injury caused by sheer stress particularly at arterial bi-furcations, narrowing or directional changes leads to intraluminal endothelial damage and dysfunction. Concomitantly, pro-inflammatory intracellular signalling pathways are recruited which lead to transcriptional up regulation of expression of growth factors (e.g. vascular endothelial cell growth factor, platelet-derived growth factor and fibroblast growth factor-2) cytokines (e.g. tumour necrosis factor-alpha and MCP-1), adhesion molecules (e.g. intracellular adhesion molecule-1 and vascular endothelial cell adhesion molecule) and chemoattractant proteins [2]. Endothelial cell damage, activation and up-regulation of adhesion molecules encourage the attraction of platelets, T-cells, and macrophages which engulf excess cholesterol transform into foam cells and help to produce fatty streaks-some of the earliest pathological sign of plaque development [3]. Endothelial dysfunction is known to impair the production and bioavailability of nitric oxide (protective against atherosclerosis) and therefore protection of these cells against damage and/or increasing the circulating nitric oxide levels using pharmacological agents could have clinical benefit for high risk candidates [4,5].

As more platelets and immune cells aggregate at a damaged region, the increased cytokine production leads to local cellular proliferation, and transmission of activating signals to the adventitial vasa vasorum which become activated and migrate through the layers of the artery to help feed the now growing plaque [6,7].

Actively growing plaques often remain stable with thick fibrous caps and contain a high proportion of smooth muscle cells. In these cases, arterial remodelling eventually results in a gradual narrowing of the lumen resulting in, patient symptoms such as angina. The mechanisms responsible for determination of the development of vulnerable unstable plaques rather than stable ones is still unknown although there is evidence for the involvement of a number of key factors, namely, oxidative stress and formation of oxidized low density lipoproteins, diabetes, high or fluctuating blood sugar levels and formation of advanced glycation end-products (AGEs), the process of inflammation and tumour-like angiogenesis.

### The importance of plaque vascularisation

Plaque angiogenesis is now accepted to have a fundamental role in the pathophysiological development of atherosclerosis, providing nutrients to the developing and expanding intima and also potentially creating an unstable haemorrhagic environment prone to rupture. The expression of intimal neovessels is directly related to the stage of plaque development, the presence of symptomatic disease and the risk of plaque rupture. In atherosclerosis, intimal neovascularization arises most frequently from the dense network of vessels in the adventitia, adjacent to a plaque, rather than from the main artery lumen. The irregular nature of blood vessel formation has been likened to tumour angiogenesis, and hence the factors responsible for their growth may be different from those seen during normal wound healing. Our previous studies and those of others have suggested that haemorrhagic, leaky blood vessels from unstable carotid plaques proliferate abnormally. These relatively large caliber but immature neovessels are poorly invested with smooth muscle cells and possess structural weaknesses which may contribute to instability of the plaque by facilitation of inflammatory cell infiltration and haemorrhagic complications Therefore; inhibition of angiogenesis might be an important target for prevention of development of active, unstable plaque lesions [7].

### Oxidative stress

The process of oxidative stress starts early in developing lesions, when inflammatory cells and/or dysfunctional mitochondra caught in the arterial wall begin to secrete reactive oxygen species (ROS) [8]. ROS are able to stimulate cellular apoptosis directly, and also to oxidize low-density lipoproteins (LDLs) [9]. Oxidised LDLs (oxLDLs) promote smooth muscle cell and macrophage gelatinase production as well as stimulating pro-inflammatory signalling pathways through tissue factor (TF), interferon regulatory factor-1 (IRF-1) and Toll-like receptor-2 (TLR2), thereby contributing significantly to plaque instability and thrombosis [10]. Similarly, particularly in diabetic patients, a combination of chronic hyperglycaemia and enhanced oxidative stress results in production and deposition of advanced glycation end-products (AGEs) in the coronary arteries, which activate intracellular processes through their receptor receptor for AGEs) (RAGE) inducing further oxidative stress, promoting inflammation via NFkappaB, and increasing ECM accumulation [11]. Reduction of plaque inflammation and oxidative stress could help to prevent plaque erosion and ultimately thrombosis.

## Current preventative and treatment strategies for CAD

Throughout Europe and the EU, patients recognized by general practitioners and within hospitals to be at risk of developing coronary artery disease (CAD) (genetic profiles, risk factor analysis, presence of symptomatic disease etc.) or with a known history of CAD, may be offered a variety of prescriptions designed to reduce the risk of first or subsequent MI. Aspirin and other anti-platelet medicines such as clopidogrel help to prevent blood clots, whilst new anti-platelet agents including prasugrel and ticagrelor have shown reduced iscaemic efficacy and greater safety for patients with acute coronary syndromes (ACS) [12]. Statins (inhibitors of HMG-CoA) are commonly prescribed to patients presenting with high total cholesterol levels or high HDL/LDL ratios when one or more related risk factors are also present. Rosuvastatin, one of the more recently distributed statins appears to have several other beneficial, preventative actions (greater than other statins) besides its lipid-lowering ability for patients with CAD. Rosuvastatin has been shown to preserve coenzyme Q10, ubiquinol and concomitantly increase HDL-C levels [13] significantly induce plaque regression by reducing plaque volume [14], and reduced vascular endothelial cell growth factor (VEGF) levels following myocardial revascularization potentially reducing plaque neovascularisation [15]. Beta (adrenoceptor antagonists)-blockers and calcium-channel blockers, reduce chronic resting heart-rate (an independent predictor of CAD mortality) and reduce blood pressure/hypertension, possibly the most important single factor associated with CAD [16], whilst angiotensin-converting enzyme (ACE) inhibitors and angiotensin II receptor blockers also reduce blood pressure and may also have a protective effect against development of atherosclerosis by ameliorating endothelial dysfunction [17].

### Limitations of current treatments

Limitations of the current treatments are clear, side-effects and long-term complications apart, the known effects of these medications show only marginal interactions with some of the major processes known to be responsible for creation of unstable plaques i.e. oxidative stress combined with inflammation and angiogenesis (with the possible exception of some types of statin which is the subject of ongoing research). As well as this, a significant amount of evidence exists that patients with none-acute cardiac disease are markedly more unlikely to receive guideline-recommended therapy [18]. Hence, the majority of at-risk individuals currently only start medication after the onset of symptoms at which time plaque development and arterial remodelling is evident or has already occurred.

## Current use of NHP in clinical practice

Diet has long been known to be a modifiable risk factor for CHD with Mediterranean diets and consumption of fish, fruit and whole grain all having beneficial effects [19]. People with diets containing high levels of substances, with anti-oxidant properties, such as vitamins E and C, and beta-carotene, tend to live longer and more healthily. A search through PubMed using the terms “natural health products” and “CAD” reveals only 21 hits, one of the most recent by Shukla et al. [20], describing profound cardiovascular protective effects of nutritional supplements such as flavonoids, olive oil, lycopene, resveratrol and soy in epidemiological and clinical studies. A far more interesting picture is seen when examining individual substances by literature analysis (see below). Most importantly, a recent study by Alherbish et al. [21] who conducted a survey on patients admitted with acute vascular disease, discovered that a wide variety of NHPs are used by patients including vitamins and minerals (73%), herbal products (20%) and amino acids/essential fatty acids etc. (35%), however, the health professionals themselves rarely included NHPs as part of their medication profile.

## Evidence for the protective properties of AGExt, Resveratrol and GTE in CVD

Very few studies have analyzed in detail or retrospectively compiled data by systematic analysis, on the effects of dietary supplements on arterial disease. One such study examined 38 published studies (clinical trials) examining the effects of NHPs on arterial stiffness measured by pulse-wave velocity [22]. The results showed that in a majority of trials, omega-3 supplementation, isoflavones, and flavonoids produced significant reductions in arterial stiffness. There was insufficient data no make conclusions in respect of other substances including herbal medicines, and garlic. Here we will focus on analysis of the data concerning three key substances to provide evidence of their potential cardio-vascular protective effects.

### Evidence for the protective properties of AGExt

In vitro studies have demonstrated that AGExt and in particular its most active components, the water-soluble cysteinyl moieties S-allylcysteine and S-allylmercaptocysteine are exceptionally powerful anti-oxidant phytochemicals, protective against oxidative stress (by scavenging reactive oxygen species and increasing superoxide dismutase levels) and inhibition of subsequent cellular damage [23,24]. AGExt was shown to suppress CD36 scavenger receptor expression on macrophages, decrease binding of nuclear proteins to a PPARγ pathway, and inhibit Dil-labelled OXLDL uptake [25]. Similarly, black AGExt demonstrated anti-inflammatory properties when administered to human umbilical vein endothelial cells (HUVEC) by preventing tumour necrosis factor-alpha (TNFα)-induced expression of adhesion molecules (VCAM-1 and ICAM-1) and inhibiting NF-κB transcription factor expression as well as reducing monocyte adhesion and ROS generation [26,27]. Hence in vitro studies demonstrate a potential use for AGExt (which has no specific side-effects and can be taken in high potentially pharmacologically effective doses) in reducing oxidative stress, inflammation, and providing endothelial protection against sheer stress damage. Can these effects prove translatable in vitro? Very few studies have investigated interactions between AGExt and pharmacologically active substances used to treat patients with CVD, although there are not thought to be any particular negative synergistic effects [28]. In 2009, Budoff et al. [29] conducted a randomized clinical trial to assess if AGExt could retard the progression of sub-clinical atherosclerosis. 65 patients were treated for one year with AGExt (250 mg) together with vitamin supplements daily. After one year, patients receiving the AGExt showed reduced coronary artery calcium and increased vascular reactivity (measured by temperature rebound). Total cholesterol, homocysteine, MDL-LDL auto-antibodies and apoB-immune complexes were decreased whilst HDL, apoB and Lp (a) were all significantly increased. In conclusion, this is the first study to demonstrate improvement in oxidative markers, vascular function and a significant reduction in the progression of atherosclerosis in association with dietary AGExt supplementation in humans.

**Figure 1:**
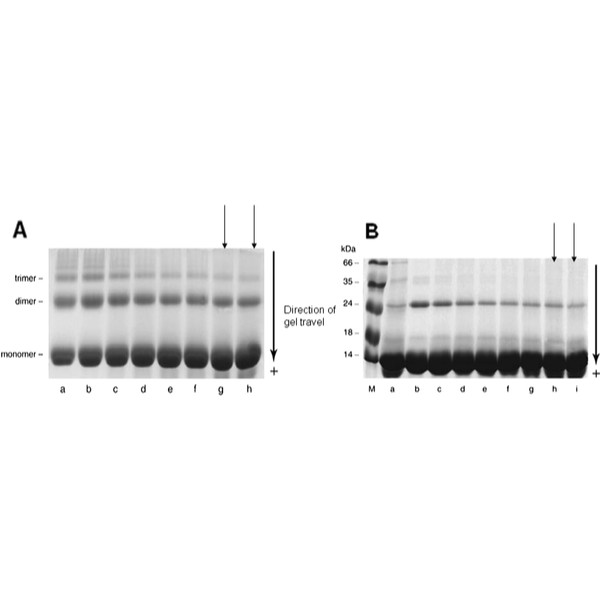
**A, AGExt (0–56 mg/ml) inhibition of dimer and trimer production in BSA exposed to high glucose (0.5 M; a-b, controls) in vitro for 21 days (c-h; 0, 7, 14, 28, 42, 56 mg/ml).B**, Lysozyme incubated in the absence (lane a) or presence of 0.5 M glucose (b–i) for 35 days and the effect of 0, 7, 14, 28, 42, 56, 70 and 84 mg/ml of AGExt on dimerization. Arrows indicate a significant reduction in dimmer and trimer formation in the presence of AGExt.

AGExt also reduced blood pressure (960 mg/day; systolic BP reduced by 10.2 mmHg; p = 0.03) [30], and inhibited production of AGEs and glycation-induced cross-linking in vitro [31] Figure 1. AGEs, produced by chronic exposure of proteins to high sugar levels are particularly prevalent in patients with diabetes. When they bind to the receptor for AGEs (RAGE), they activate cellular signalling pathways associated with inflammation, generation of oxidative stress and ROS and are associated with premature development of atherosclerotic diabetic lesions, rich in leukocyte infiltrates [32,33]. Components of AGExt (N-acetylcysteine) and AGExt itself have been shown to reduce expression of RAGE, endothelial cell adhesion molecules, NF-κB, MDA, ROS generation and matrix metalloproteinases culminating in plaque stabilization in apoE-deficient mice [34].

### Resveratrol has strong anti-oxidative and anti-inflammatory properties

**Figure 2:**
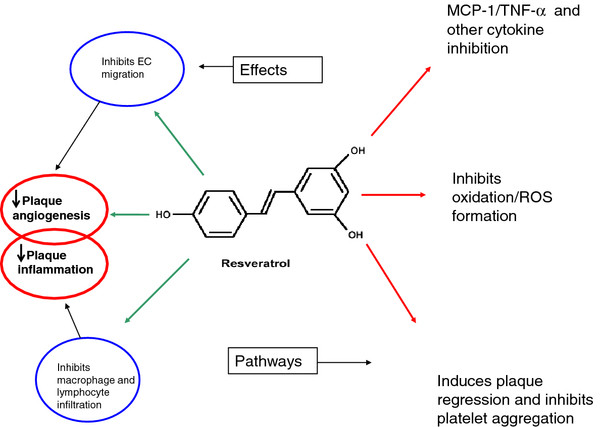
**Schematic showing the structure of resveratrol and some of its most potent effects on intracellular signalling associated with prevention of EC activation and unstable plaque development.** Its potent anti-oxidant, anti-inflammatory and EC protective effects are included here and are thought to be the main reason for its cardio-protective effects and ability to inhibit plaque de-stabilization.

*Trans*-resveratrol, a polyphenol, and the major grapevine phytoalexin (3,5,4´-trihydroxystilbene; Figure 2) has attracted a lot of attention in the last couple of years due to its extremely potent anti-oxidative and anti-inflammatory capacity and its potential use in the treatment of vascular disease and prevention or attenuation of atherosclerosis [35]. This group of anti-oxidants is thought to be responsible for the ‘French paradox’ where a low mortality rate for CAD exists in the population despite their diet of high fat and smoking [36]. In vitro and in vivo studies have identified a multitude of potentially protective effects of this compound. For example, Balestrieri et al., [37], demonstrated that resveratrol or indeed red wine alone could significantly prevent TNF-α-induced reduction in endothelial cell progenitor (EPC) cell number at physiological concentrations which could positively impact on re-endothelization of damaged vessels. Resveratrol was also shown to block Ca^2+^ influx in thrombin-stimulated platelets and to block ADP, collagen and thrombin induced platelet aggregation through a pathway which may involve inhibition of thromboxane A2 [38]. Similarly, resveratrol was shown to inhibit human platelet aggregation stimulated by collagen and concomitantly induce platelet apoptosis through activation of caspases-9, 3, and 8 as well as gelsolin and actin cleavage [39]. Prevention of aggregation coupled with apoptosis may represent an important mechanism for reduction in thrombotic events.

Resveratrol also inhibited both endothelial cell migration and MCP-1-induced monocyte cell chemotaxis, which could potentially reduce neointimal vascularisation and monocyte recruitment into a growing plaque [40]. Regarding inflammation, roscovitine is a potent inhibitor of NF-κB activation, other inflammatory gene expression (e.g. IL-6, IL-8), endothelial cell adhesion molecule expression including ICAM-1 and monocyte adhesiveness to endothelial cells (and recruitment into the artery wall) in an Akt-dependent manner [35,41]. Resveratrol also inhibits cyclooxygenase and hydroperoxidase functions thereby reducing inflammation even more potently [42]. Using an apoE knockout mouse model, Norata et al. [43] showed that animals fed with a supplement containing cathechin, caffeic acid and resveratrol, had significantly smaller plaques after 8 weeks (36–40%) in the aortic sinus and ascending aorta mainly due to a reduction in inflammatory infiltration and expression of MCO-1, MIP-1α, MIP-1β, CCR1, CCR2 and ET1 in the vascular wall. Similarly, Kim et al. [44] showed that oral administration of resveratrol suppressed intimal hyperplasia in a wire-injured femoral artery mouse model. Their further examination of cultured smooth muscle cells revealed inhibition of PDGF-induced ROS and cell proliferation as well as increased expression of Nrf2 and anti-oxidant response element reporter activity associated with HO-1 induction. This data was in agreement with work performed by Brito et al., [45], who showed that resveratrol could inhibit oxLDL-induced smooth muscle cell proliferation via inhibition of the PI3K/Akt/mTOR/p70S6K pathway.

Endothelial cell function is known to be improved in the presence of resveratrol through potentiation of endothelial NO synthase (eNOS) via activation of PPARα and SIRT1 in vascular endothelial cells and diabetic rats in vivo [38,46]. In monocytes from diabetic patients resveratrol, counteracted the pro-atherosclerotic effects of high glucose levels by reducing super-oxide production through activation of mitochondrial signalling pathways involving SIRT1-FOXO. E-selectin was also down-regulated [47]. Studies with type-1 diabetic rats have shown that treatment with resveratrol (10 mg/kg) prevented impairment in eNOS and nNOS-dependent vasodilation in cerebral blood vessels. eNOS and nNOS were also increased whilst super-oxide dismutase was reduced suggesting a restorative function for vascular tissue and oxidative stress [48]. Nitric oxide regulates vascular tone, causes endothelium-dependent vasodilation, and decreases platelet aggregation. In addition it acts as an anti-oxidant, anti-proliferative and anti-inflammatory molecule giving it a key role in the inhibition/prevention of atherosclerosis [49]. A multitude of studies have also shown a significant reduction in oxidative stress in a variety of cardiovascular-implicated cells including blood mononuclear cells (decreased malondialdehyde concentration), human platelets (reduction in peroxynitrite-induced oxidation), super-oxide scavenging in damaged rat myocardial tissue and inhibition of LDL oxidation ([50] for a review).

Other studies show the enormous potential clinical applications of this compound from demonstrations of its complete lack of toxicity in humans even at high doses, to its lipid lowering capability in mammalian models and general ‘reduction in modifiable risk factors’ [51,52] and protection of cardiomyocytes against apoptosis [53].

### Flavanols and other compounds in green and black tea extracts

**Figure 3:**
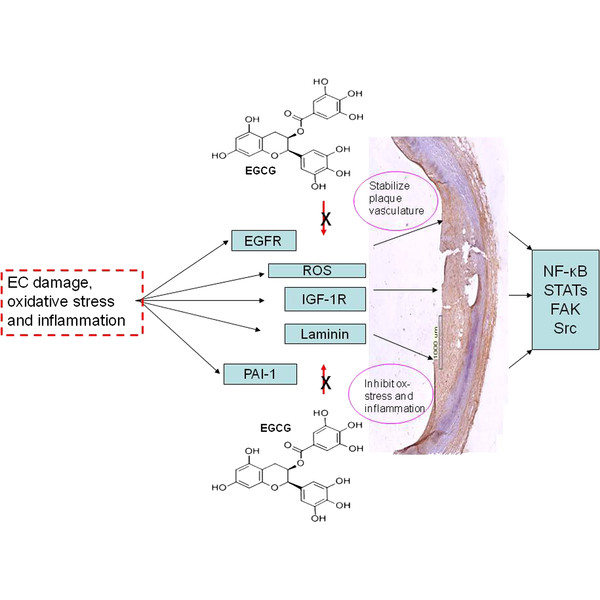
**Representative diagrams showing the interaction between EGCG and important signalling pathways associated with nuclear response gene activation and subsequent inflammation and oxidative stress.** Key: EGFR, Endothelial Growth Factor Receptor; ROS, Reactive Oxygen Species; IGF-1R, Insulin Growth Factor-1 Receptor; PAI-1, Plasminogen Activator Inhibitor-1; NF-κB, Nuclear Factor-kappa-B; FAK, Focal Adhesion Kinase.

Green and black tea and green and black tea extracts are a potent source of flavanols (catechins), phenolic acids and flavonoids, with the catechin (−)-epigallocatechin-3-gallate (EGCG; Figure 3) perhaps being the most abundant [54]. Studies have shown that these substances delivered as an extract from de-caffeinated tea (455 mg/day) could reduce hemodialysis-induced ROS generation and pro-inflammatory cytokine expression in patients [55]. Cross-sectional, randomized controlled and prospective population studies have shown that tea intake and/or increased dietary tea flavonoids reduced the risk of cardiovascular disease, with consistent data demonstrating enhancement of NO production and concomitant improvement of endothelial function as well as reduction in total cholesterol levels and LDL cholesterol [56]. In one particular population-based prospective cohort study, (the Ohsaki study), 40,530 persons from Northern Japan were enrolled, and data demonstrated an inverse relationship between CVD mortality and tea consumption [57]. Ras et al. [58] showed by meta-analysis of nine studies that tea consumption was directly associated with increased (40%) flow-mediated dilation of the brachial artery (a measurement of endothelial function), and Tinahones et al., [59] using the same methodology also showed concomitant reduction in oxLDL levels in patients taking green tea extract supplements demonstrating potential protection against CVD.

These polyphenols were also shown to significantly reduce serum glucose and lipid peroxidation levels in alloxan diabetic rats [60]. In vivo studies have shown that these green tea extracts appear to have insulin-like activities, with EGCG inhibiting intestinal glucose uptake via the sodium-dependent glucose transporter SGLT1 [61]. In vitro, tea extracts were shown to inhibit endothelial cell plasminogen activator inhibitor-1 (PAI-1; inhibits fibrinolysis and increases the risk of thrombolysis) through a pathway involving PI-3 K/Akt, again, suggesting they may contribute to cardiovascular protection [62]. Other studies have demonstrated that green tea extracts reduced plasma triglycerides, insulin resistance and oxidative stress in insulin-resistant Wistar rats which suggests a potential protective role against diabetes-associated premature development of CVD in patients [63].

## Should NHP be prescribed as main-line drugs and/or supplements for patients at risk of MI?

A substantial amount of data including that provided above strongly suggests that either alone or possibly in combination (in the form of a super pill), substances derived from natural health products such as EGCG, resveratrol and s-allylcysteine, may have important cardiovascular health benefits. Although much of the data is based on work carried out in vitro (mostly to determine mechanisms of action) and using animal models, a significant amount of recently reported data from clinical trials now suggests these products prescribed alone or in combination may prove to be important alternative or additional main-line prescription drugs. Many of these bioactive phytochemicals have synergistic effects making multi-extract combinations more efficient for targeting more than one target and also meaning that lower oral doses can be used. For example, Baile et al. [64] demonstrated that a mixture including resveratrol and quercetin was able to reduce adipogenesis in ovariectomized rats, and a number of studies have showed increased anti-tumour activity of polyphenolic mixtures. However, so far, there are very few studies examining the potential benefits of these mixtures in protection against or amelioration of CAD and secondary complications.

Hollman et al., [65] recently concluded that whilst in vitro studies, in vivo models of disease and retrospective studies strongly suggest that polyphenol-rich products could have beneficial effects, and that circulating markers of CVD such as oxLDL, total anti-oxidant capacity, F2-isoprostanes and ox-LDL) are often reduced, prospective studies are lacking, and causal relationships have not yet been reliably found. The authors go on to suggest that a direct effect of these bioactive compounds is highly unlikely as metabolism during ingestion would result in lower serum circulating levels than those of other existing anti-oxidants. Furthermore, much more detailed work needs to be applied to understanding fully the mechanisms of action of these substances and how each interacts with the most important factors (inflammation, oxidative stress, angiogenesis, diabetes and hypertension) associated with early-medium levels of arterial damage, plaque development and ultimately symptomatic CAD. Smoliga et al., [66] describes using resveratrol as an example the lack of understanding of pharmokinetics of this compound in human metabolism, long-term toxicity studies and drug-drug interactions, all of which must be established prior to consideration or justification of its use in routine clinical practice. The situation would become even more complex in the design of a super pill composed of several ingredients targeting more than one anti-atherosclerotic pathway. In vitro studies demonstrated that higher doses of EGCG were toxic to rat hepatocytes (100–500 μg/ml) and caused pancreatic β-cell damage in streptozotocin-induced diabetic rats (5 mg/kg/day) [67,68], confirming the importance of further studies.

Nevertheless, the striking potential benefits of the three substances we have reviewed as well as others originating from for example soy, olive oil and cocoa should be continued to be investigated, with justification, as the world of vascular biology and in particular the devastating disease of atherosclerosis is desperately in need of identification and approval of novel therapeutics. Therefore, at the present time this group of natural product-derived substances may represent the best new pharmacological preventative/protective treatment strategy. So far, the numbers of in vivo studies examining the active ingredients of these substances are still limited, the major active molecules remain to be identified and synergistic effects studied in detail before we are ready to contemplate realistic clinical trials in association with detailed comparisons with existing pharmacological agents used for treatment of the major CAD/cerebrovascular risk factors.

## Competing interests

The authors declare that they have no competing interests

## Author’s contributions

MS and GM drafted the manuscript; LB, NA and QW edited and provided additional sections. All authors read and approved the final manuscript.
